# MiR-4298 and lncKRTAP5-6-3 regulated Cathepsin D expression through ERK-MAPK signaling pathway in chronic UVB-damaged HaCaT cells

**DOI:** 10.3389/fmed.2024.1485224

**Published:** 2025-01-13

**Authors:** Xinling Chen, Feng Zhou, Yao Lin, Yue Xia, Jie Zhang, Wenyi Hou, Yu Sun, Wei Lai, Yue Zheng

**Affiliations:** ^1^Department of Dermato-Venereology, The Third Affiliated Hospital, Sun Yat-sen University, Guangzhou, Guangdong, China; ^2^Sun Yat-sen Memorial Hospital, Sun Yat-sen University, Guangzhou, Guangdong, China; ^3^Nanfang Hospital, Southern Medical University, Guangzhou, Guangdong, China

**Keywords:** chronic UV damage, miR4298, lncRNA, ERK-MAPK signaling pathway, Cathepsin D

## Abstract

**Objective:**

MiRNAs and lncRNAs are important regulators in the process of skin photoaging. In this study, we investigated the expression changes and interactions between miR4298 and lncKRTAP5-6-3 in chronically UVB-damaged human keratinocyte cell line (HaCaT) cells and explored miR4298-MAPK/ERK signaling pathway-Cathepsin D-lncKRTAP5-6-3 mechanisms in photoaging cells.

**Methods:**

HaCaT cells were irradiated with 12 mJ/cm^2^ UVB once a day for 7 days. miR-4298 mimics and miR-4298 inhibitors were transfected into HaCaT cells by lipo3000 transfection reagent, and the HaCaT cells were divided into three groups: blank control group; UVB-damaged group; and UVB damage+miR-4298 regulation (overexpression or inhibition) group. The expression levels of miR4298 and lncKRTAP5-6-3 were quantitatively analyzed using RT-PCR, while the expression of Cathepsin D and MAPK/ERK signaling pathway proteins was detected using Western blot.

**Results:**

After 7 consecutive days of UVB irradiation, the expression of miR-4298 decreased by 0.64 ± 0.06 (*P* < 0.001) compared to the un-irradiated HaCaT cells, and the expression of the KRTAP5-6-3 decreased by 0.80 ± 0.13 (*P* < 0.001) compared to the control group. The expression of p-ERK signaling was increased by 0.9437 ± 0.1186 (*P* < 0.0001), and Cathepsin D was decreased by 0.6163 ± 0.075 (*P* < 0.0001). In HaCaT cells transfected with miR-4298 mimics and then irradiated by UVB for 7 days, the expression of lncKRTAP5-6-3 was increased to 0.5114 ± 0.1438 (*P* < 0.05)-fold, and the phosphorylation level of ERK signaling was decreased by 0.3880 ± 0.1185 (*P* < 0.01), while Cathepsin D expression was increased by 0.2617 ± 0.0749 (*P* < 0.0001) compared to the UVB-damaged group. In HaCaT cells transfected with miR-4298 inhibitors and then irradiated by UVB for 7 days, lncKRTAP5-6-3 was decreased by 0.1697 ± 0.1383, the phosphorylation level of ERK signaling was increased by 1.096 ± 0.7836 (*P* < 0.05), while Cathepsin D expression was decreased by 0.05197 ± 0.24827 compared to the UVB-damaged group.

**Conclusion:**

The synergistic effects of miR4298 and lncKRTAP5-6-3 play important roles in chronic UVB-damaged HaCaT cells by regulating the MAPK/ERK signaling pathway and Cathepsin D expression. This study presents novel targets for intervening in chronic ultraviolet damage (photoaging) skin and UV-related dermatoses.

## 1 Introduction

Repetitive and chronic ultraviolet (UV) radiation is the most important factor in skin aging (1). Repeated chronic UVB radiation could cause various skin diseases and cutaneous tumors (2–4).

Previous studies revealed that chronic UV radiation can lead to abnormal accumulation of glycosylation end products (AGEs) in skin cells (5, 6). The expression level and activity of Cathepsin D (CTSD), a hydrolytic protein active in the acidic environment of the lysosome (7), were decreased and negatively correlated with the accumulation of AGEs in the skin. Furthermore, the content of CTSD in the skin could be increased by *in vitro* CTSD gel application, which helps repair skin damage (8, 9). However, CTSD, a protein with a large molecular weight, was inefficient in entering the skin when administered *in vitro*. Thus, more stable and efficient regulatory measures targeting CTSD in photoaging skin need to be explored.

LncKRTAP5-6-3, lncRNAs encoded by keratin-associated protein (KRTAP) genes, were found in hair shafts as an assembly of keratin bundles (10). KRTAP5-5 also could regulate cytoskeletal and vascular infiltration (11). Our previous studies had identified that the expression of lncKRTAP5-6-3 was downregulated in the detection of fibroblast lncRNA expression profiles after chronic UVA irradiation, and GO analysis suggested that CTSD was a downstream target gene of lncKRTAP5-6-3, which could regulate the expression of Cathepsin D protein (12). Recent studies have revealed that lncRNAs might act as sponges for miRNAs. The compound style of miRNAs-lncRNAs could not only take part in altering the lncRNAs expression but also regulate its downstream genes and proteins expression (13).

MiR4298, differentially expressed in human myeloma, glioblastoma, and persistent atrial fibrillation, is a promising novel biomarker (14–16) and has been used in clinical treatments and prognosis assessments (17, 18). In further studies, it has been found that the stress-induced miR-31-CLOCK-ERK pathway is a key driver and therapeutic target for skin aging (19). The study by Gao et al. showed that exosomes overexpressing miR-1246 could protect against UVB-induced photoaging by inhibiting the TGF-β/Smad and MAPK/AP-1 pathways (20). However, studying the oncogenic role of miR1246 more extensively, it turned out that it mainly regulates the Wnt/β-catenin signaling pathway (21–28). A study on cone cornea showed that miR4298 is mainly involved in NF-kB signaling and MAPK signaling pathways (29). However, the mechanism of miR4298′s role in chronic UVB-damaged cells remained unclear.

In this study, we investigated the regulation of miR-4298 on IncKRTAP5-6-3 in chronic UVB-damaged HaCaT cells, explored further the regulatory mechanisms of the ERK-MAPK signaling pathway, and involved biological functions of Cathepsin D.

## 2 Materials and methods

### 2.1 Materials

Human keratinocyte cell line (HaCaT) cells were purchased from Wuhan Procell Life Science & Technology Co., Ltd. The UVB irradiation machine (SH4B) was purchased from Sigma-Aldrich. The design and synthesis of miR-4298 mimics and inhibitors were performed by Suzhou GenePharma Co., Ltd. MiR4298 mimics are small double-stranded RNA molecules designed to mimic the endogenous mature miR4298 sequence based on the miR4298 mature sequence. MiR4298 mimics consist of a sequence aligned to the miR4298 mature sequence and a complementary sequence. MiR4298 inhibitor is a single-stranded 21–23 nt 2′-methoxy-modified RNA oligonucleic acid that effectively inhibits the function of endogenous mature miR4298. The design sequence of miR4298 mimics and inhibitors is listed in Table 1. The lipo3000 reagents for transfection were purchased from Thermo Fisher Scientific. The primers used in RT-PCR were synthesized by Beijing Ruibo Xingke Biotechnology Co., Ltd. The primary antibodies used in the Western blot experiment were purchased from Abcam (Shanghai) Trading Co., Ltd.

**Table 1:** Nucleotide sequences used in the study.

**Name**	**Sequence(5^′^-3^′^)**
MiR4298 mimics	CUGGGACAGGAGGAGGAGGCAG
MiR4298 inhibitors	CUGCCUCCUCCUCCUGUCCCAG
Mimic NC	UUCUCCGAACGUGUCACGUTT
Inhibitors NC	CAGUACUUUUGUGUAGUACAA
MiR-4298-F	ACACTCCAGCTGGGCTGGGACAGGAGGAGGAG
MiR-4298-R	CTCAACTGGTGTCGTGGA
MiR-4298-RT	CTCAACTGGTGTCGTGGAGTCGGCAATTCAGTTGAGCTGCCTCC
U6-F	CTCGCTTCGGCAGCACA
U6-R	AACGCTTCACGAATTTGCGT
U6-RT	AACGCTTCACGAATTTGCGT
KRTAP5-6-3-F	GGACCAAGACCCTGCAATGA
KRTAP5-6-3-R	CCTCCCTGATATGCCCCGA
GAPDH-F	GGGAAACTGTGGCGTGAT
GAPDH-R	GAGTGGGTGTCGCTGTTGA

### 2.2 Cell culture

HaCaT cells were cultured in MEM (with NEAA) medium (Procell), supplemented with 15% fetal bovine serum (FBS, Gibco) and 1% antibiotic solution (penicillin 100 U/mL and streptomycin 100 μg/mL), at 37°C in an atmosphere of 95% saturated humidity and 5% CO_2_. HaCaT cells were grown adherently, the culture medium was changed every 2–3 days, and the cells were trypsin digestion and subculturing when the cells were grown to 80%−90% confluent. HaCaT cells at the logarithmic growth stage were selected for subsequent experiments.

### 2.3 Chronic UVB damaging HaCat cell model

Well-growing HaCaT cells were seeded into six-well plates at a density of 40%–50% 1 day in advance, and UVB radiation was started 24 h later. The cells were divided into two groups: (1) the control group and (2) UVB damaging group. Before irradiating the cells, the medium was removed and the cells were washed 1–2 times with PBS. Then, 1 mL of PBS was added, and the cells were irradiated at a height of ~4 cm. For the UVB damaging group, the UVB irradiation dose was 12 mJ/cm^2^/time (1 min), once a day for 7 consecutive days. Our initial experiments revealed that daily irradiation at this dose for 7 consecutive days decreased the viability of HaCaT cells by ~50% (30).

### 2.4 Transfection of miR-4298 mimics

HaCaT cells were divided into three groups: (1) blank control group—normal HaCaT cells transfected with a blank vector; (2) UVB damaging group—chronic ultraviolet-damaged HaCaT cells transfected with a blank vector; (3) UVB damaging and miR-mimics group—chronic UV-damaged HaCaT cells transfected with miR-4298 mimics. The miR-4298 mimics were transfected into HaCaT cells using lipo3000 (Thermo Fisher) according to the manufacturer's instructions. HaCaT cells were inoculated in six-well plates and transfected at a density of 70%−80%, usually 24 h after inoculation. Then, 4 μL lipo3000 and 4 μL miR-4298 mimics were added to each well, and the lipo3000 and miR-4298 mimics were diluted with an equal amount of opti-MEM medium (Gibco), mixed, and allowed to stand at room temperature for 15 min before addition. UVB radiation was started 24 h after the first transfection. The second transfection was performed on the 4th day after radiation.

### 2.5 Transfection of miR-4298 inhibitors

HaCaT cells were divided into three groups: (1) blank control group—normal HaCaT cells transfected with a blank vector; (2) UVB damaging group—chronic ultraviolet-damaged HaCaT cells transfected with a blank vector; (3) UVB damaging and miR-inhibitor group—chronic UV-damaged HaCaT cells transfected with miR-4298 inhibitors. The miR-4298 inhibitors were transfected via the same technical means.

### 2.6 MicroRNA extraction and reverse transcription

A microRNA extraction kit (HaiGene Biotech Co., Ltd) was used for miRNA extraction. Cells were lysed and poured into miRNA adsorption columns according to the operating instructions, centrifuged and washed once each with 75% isopropanol and anhydrous ethanol, and finally eluted with RNase-free TE buffer, and the eluent was the extracted cellular miRNA, which could be reverse transcribed for RT-qPCR or cryopreserved at −80°C after the concentration was determined. The microRNA rapid reverse transcription kit (Shanghai Yishan Biotechnology Co., Ltd) was used for reverse transcription of miRNA. Next, 1 μL DNAzyme was added to 10–20 ng of miRNA, and the reverse transcription system was prepared according to the manufacturer's instructions. The reaction conditions were 37°C for 15 min and 42°C for 10 min. cDNA strands were obtained after the reaction was completed.

### 2.7 RT-PCR

Total RNA from the samples was extracted using the RNA Extraction Kit (HaiGene Biotech Co., Ltd). The RT-PCR process was divided into two stages: cDNA synthesis and PCR. cDNA was synthesized using the PrimeScript RT reagent kit (TaKaRa). The reaction conditions were 37°C, 15 min; 85°C, 5 s; 4°C, ∞. cDNA amplification was performed on a LightCycler 480 using the TB Green Premix Ex Taq II kit (TaKaRa). The reaction conditions were as follows: (1) pre-denaturation (1 cycle): 95°C for 30 s; (2) PCR reaction (40 cycles) 95°C for 5 s, 60°C for 30 s; (3) thawing (1 cycle): 95°C for 5 s, 60°C for 60 s; and (4) cooling (1 cycle): 50°C for 30 s. Each sample was tested three times. The average 2^−ΔΔCt^ values were calculated and selected. Glyceraldehyde-3-phosphate dehydrogenase (GAPDH) was used for total RNA normalization and U6 small nuclear 1(U6) for microRNA normalization. The primer sequences used for RT-PCR are listed in Table 1.

### 2.8 Western blot

The protein extraction kit was used to extract total cellular protein (KeyGen Biotech). Protein concentration measurement was done using the BCA Protein Quantitation Kit (Thermo). Equal amounts of protein were separated using a 10% SDS-PAGE gel before being transferred to a PVDF membrane. After blocking the membranes with skim milk (for the phospho-proteins, we chose bovine serum albumin [BSA]), they were incubated with primary antibodies overnight at 4°C. Following subsequent incubation with a secondary antibody, the blots were scanned using a fully automated chemiluminescence image analysis system (Tanon), and enhanced chemiluminescence (Affinity) was used to visualize them. The intensity of bands was measured using the ImageJ software. All values were normalized to the GAPDH value. The primary antibodies used in this research were as follows: GAPDH (Abcam, 1:3000), ERK (Abcam, 1:1000), phospho-ERK (Abcam, 1:1000), p38 (Abcam, 1:1000), phospho-p38 (Abcam, 1:1000), JNK (Abcam, 1:1000), phospho-JNK (Abcam, 1 μg/mL), Smad2 (Abcam, 1:2000), TGF-beta (Abcam, 1:1000), and Cathepsin D (Abcam, 1:5000).

### 2.9 Statistical analysis

Each experiment was performed in triplicate and repeated at least three times. SPSS 25.0 and GraphPad 9.0 software were used for the statistical analysis. The data were presented as mean ± (SD). A one-way ANOVA analysis or *t*-test analysis was employed to assess the differences between groups for normal distribution data. Concerning expression levels of folds or ratios, the Kruskal–Wallis test was used for calculating statistical significance. A *P*-value of < 0.05 was regarded as statistically significant.

## 3 Results

### 3.1 Chronic UVB-damaged HaCaT cell model establishment

**Figure 1:**
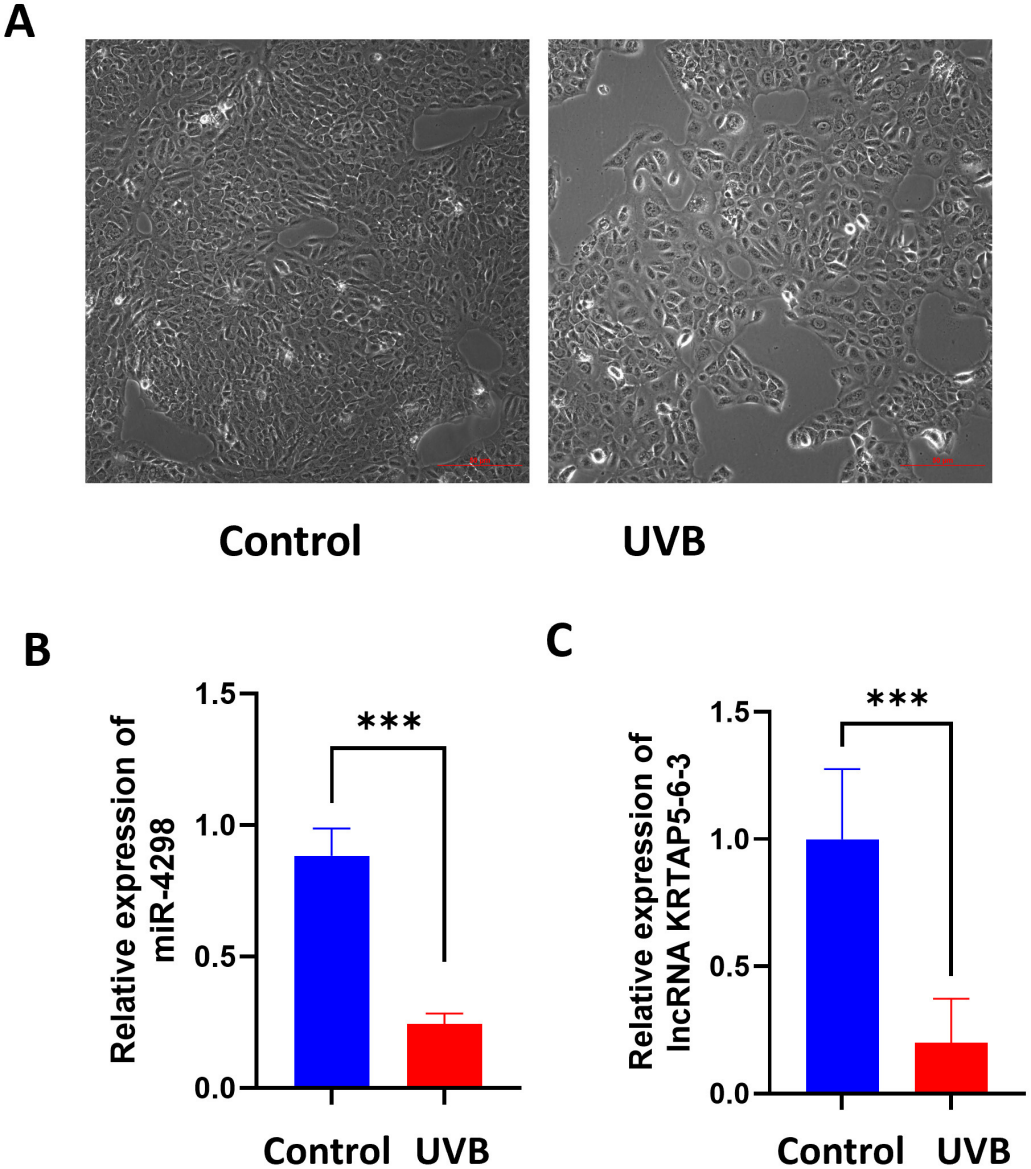
Cell morphology of each group after 7 consecutive days of UVB irradiation (Magnification: 10×). Relative expression levels of miR-4298 and its downstream lncRNA (KRTAP5-6-3) were compared between the control and UVB groups. **(A)** Compared to the control group, the cells grew at a slower rate after UVB irradiation, they became irregular in morphology and showed larger nuclei. (**B)** RT-PCR results of miR4298 expression levels in different cell groups (***P < 0.001 vs. control). (**C)** RT-PCR results of lncKRTAP5-6-3 expression levels in different cell groups (***P < 0.001 vs. control).

After UVB irradiation (12 mJ per day for 1 week), the cell shape became irregular, the nucleus enlarged, and the cytoplasmic granules were increased (shown in Figure 1A).

### 3.2 MiR-4298 and lncRNA (KRTAP5-6-3) were deceased in UVB-irradiated HaCaT cells

After 7 days of UVB irradiation, miR-4298 decreased by 0.64 ± 0.06 (*P* < 0.001) compared to the un-irradiated HaCaT cells via RT-PCR (shown in Figure 1B); meanwhile, the expression of KRTAP5-6-3 decreased by 0.80 ± 0.13 (*P* < 0.001) (shown in Figure 1C).

### 3.3 MiR-4298 regulated KRTAP5-6-3 expression

**Figure 2:**
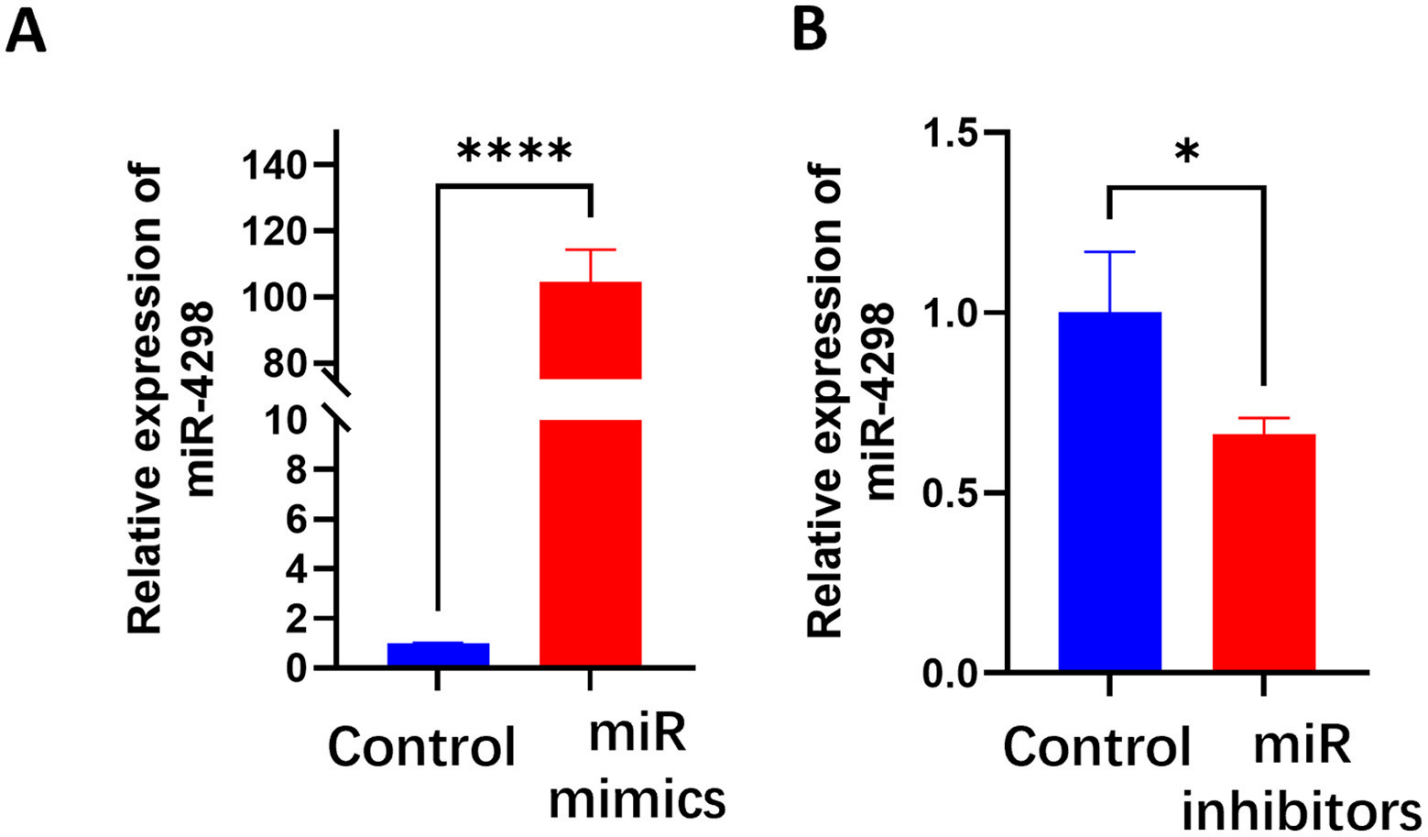
Transfection efficiency of miR-4298 by RT-PCR. **(A)** After HaCaT cells were transfected with miR-4298 mimics, the expression of miR4298 was increased by 112.9 ± 6.176-fold compared to the un-mimics group (****P < 0.0001). **(B)** Transfection with miR-4298 inhibitors decreased the expression level of miR4298 in HaCaT cells by 0.3600 ± 0.1228 compared to the un-inhibitor group (*P < 0.05).

In HaCaT cells transfected with miR-4298 mimics, the expression of miR4298 was increased by 112.9 ± 6.176 (*P* < 0.0001)-fold compared to the un-mimic group (shown in Figure 2A). Transfection with miR-4298 inhibitors decreased the expression level of miR4298 by 0.3600 ± 0.1228 (*P* < 0.05) compared to the un-inhibitor group (shown in Figure 2B).

**Figure 3:**
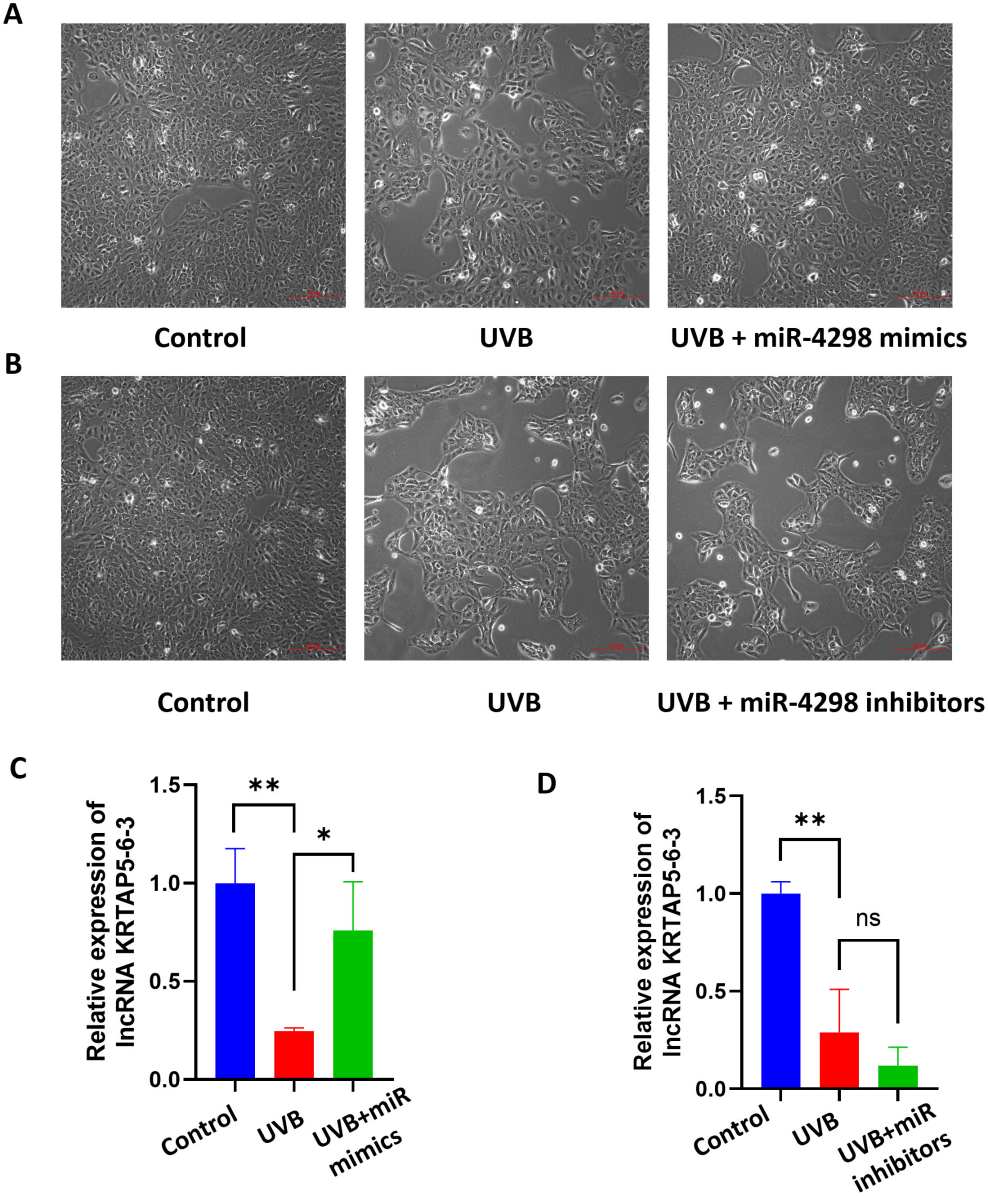
Cell morphology and relative expression levels of lncKRTAP5-6-3 in different treatment groups after transfection with miR4298 (Magnification: 10×). **(A)** After transfecting the cells with miR4298 mimics, the extent of UVB damage was significantly reduced, and cellular senescence gradually improved. **(B)** Conversely, suppressing the expression of miR4298 led to more severe cellular damage, slower cell growth, larger size, and more irregular morphology. **(C)** Differential expression of lncKRTAP5-6-3 in different cell groups after transfection with miR4298 mimics. **(D)** Differential expression of lncKRTAP5-6-3 in different cell groups after transfection with miR4298 inhibitors (*P < 0.05, **P < 0.01, ns: no significance).

In UVB+miR-4298 mimics group, the cell shape was comparatively regular, there was no overt nucleus expansion (shown in Figure 3A), and the lncKRTAP5-6-3 expression was increased by 0.5114 ± 0.1438(*P* < 0.05) (shown in Figure 3C). In UVB+miR-4298 inhibitor group, more obvious changes of cell damage phenotypes were observed (shown in Figure 3B), and lncKRTAP5-6-3 expression was decreased by 0.1697 ± 0.1383 (shown in Figure 3D).

### 3.4 MiR-4298 regulated MAPK/ERK signaling pathway

**Figure 4:**
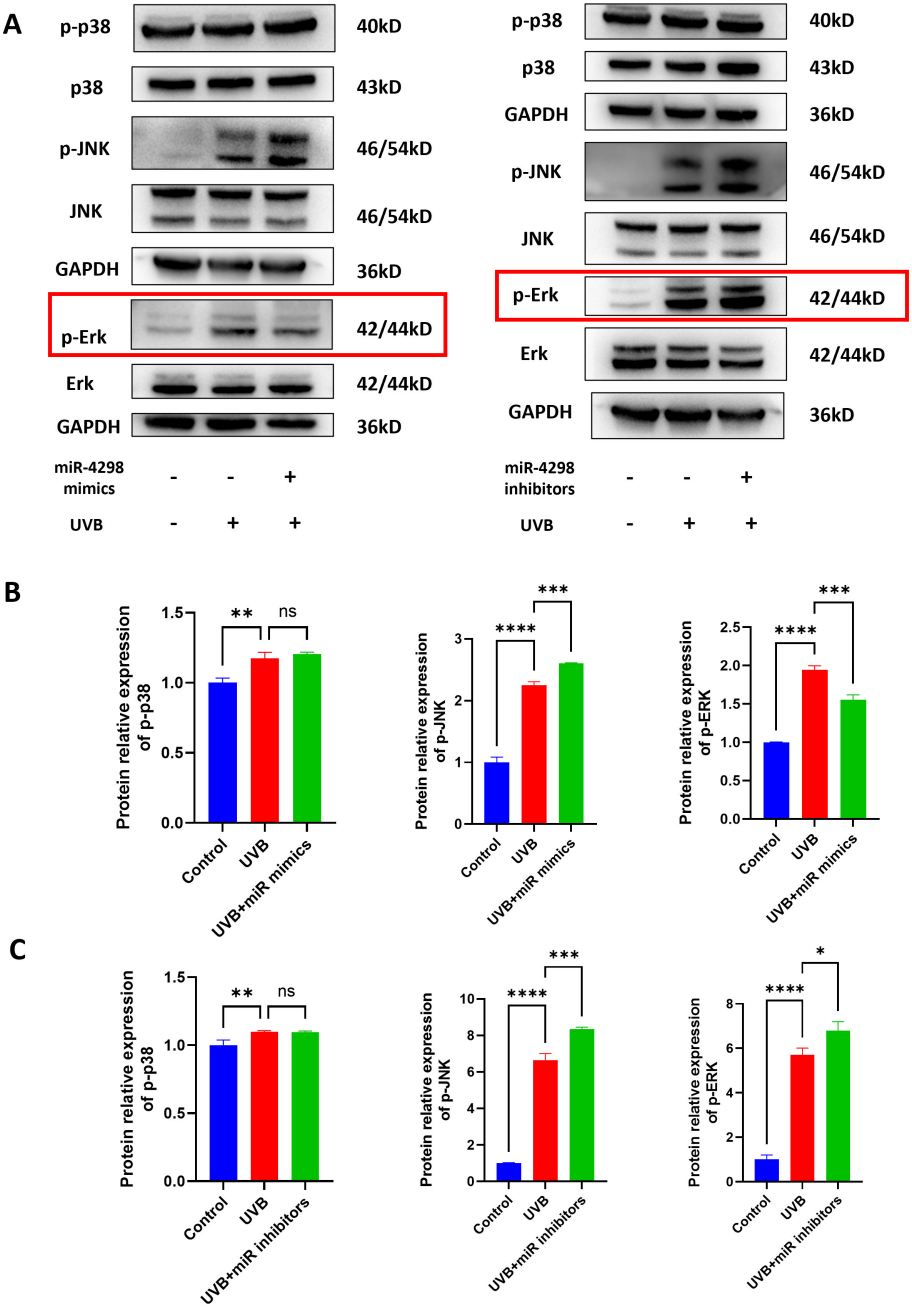
Effect of miR4298 on MAPK/p38, MAPK/JNK, and MAPK/ERK signaling pathway proteins. **(A)** Western blot results of p–p38, p-JNK, and p-ERK in different cell groups. **(B)** Statistical display of protein gray value in different cell groups after transfection with miR4298 mimics. **(C)** Statistical display of protein gray value in different cell groups after transfection with miR4298 inhibitors (*P < 0.05, **P < 0.01, ***P < 0.001, and ****P < 0.0001, ns: no significance).

**Figure 5:**
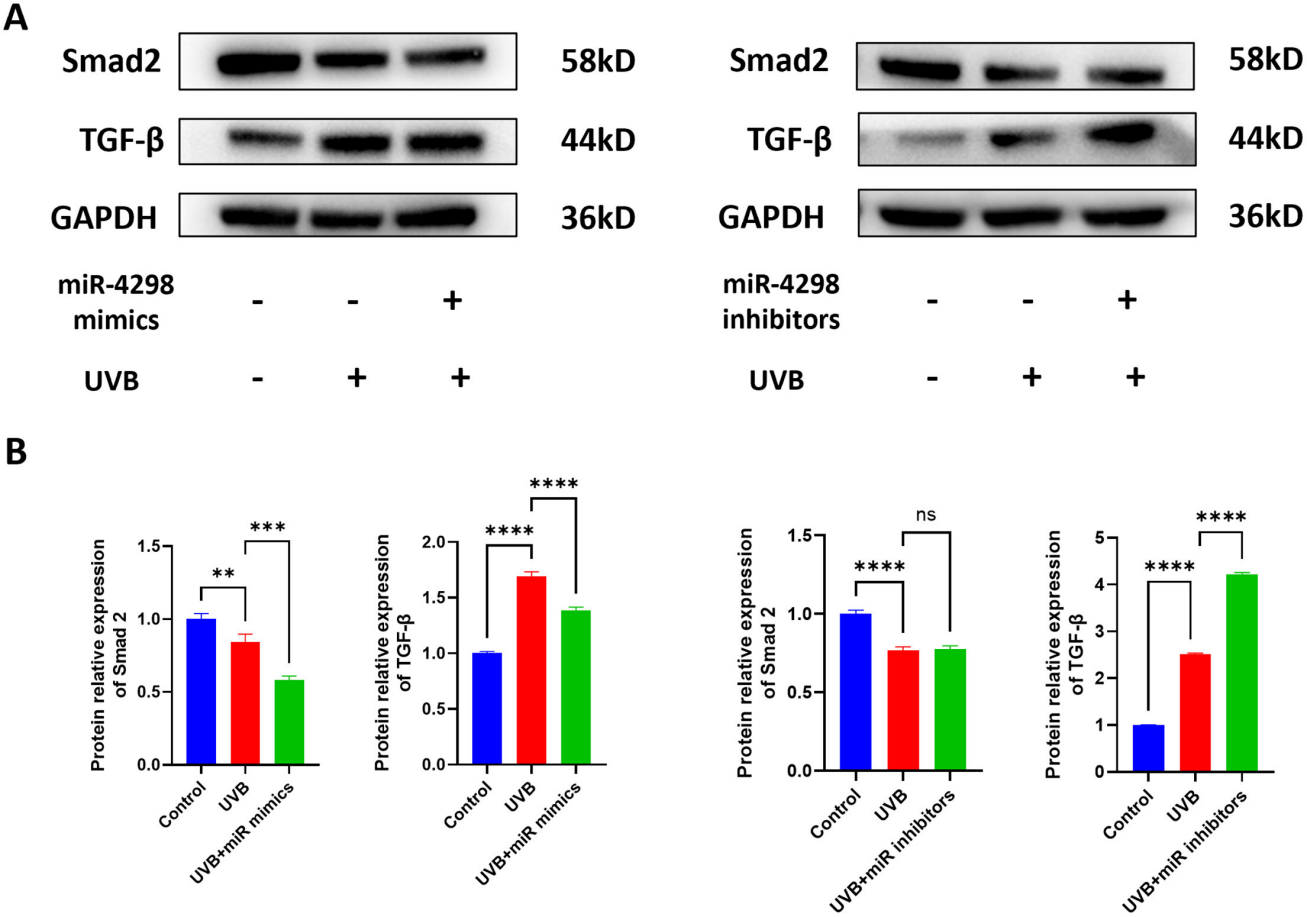
Effect of miR4298 on TGF-β/Smad2 signaling pathway proteins. **(A)** Western blot results of TGF-β and Smad2 in different cell groups. **(B)** Statistical display of protein gray value in different cell groups after transfection with miR4298 mimics. **(C)** Statistical display of protein gray value in different cell groups after transfection with miR4298 inhibitors (**P < 0.01, ***P < 0.001, and ****P < 0.0001, ns: no significance).

The results of Western blotting showed that UVB irradiation upregulates TGF-beta/Smad 2 signaling pathways and MAPK phosphorylation, including those of ERKs, JNKs, and p38 kinase (shown in Figures 4A, 5A). In UVB-irradiated HaCaT cells, the expression of p-p38, p-JNK, and p-Erk was increased by 0.1738 ± 0.081 (*P* < 0.01), 1.249 ± 0.152 (*P* < 0.0001), and 0.9437 ± 0.1186 (*P* < 0.0001), respectively. Overexpression of miR4298 changed the expression of p-JNK and p-Erk by −0.3574 ± 0.1518 (*P* < 0.001) and 0.3880 ± 0.1185 (*P* < 0.01), while p-p38 was not significantly changed (0.03029 ± 0.081, *P* > 0.05) (shown in Figure 4B).

The expression of p-p38, p-JNK, and p-Erk was increased by 0.09775 ± 0.05871 (*P* < 0.01), 5.646 ± 0.54 (*P* < 0.0001) and 4.704 ± 0.784 (*P* < 0.0001), respectively, of HaCaT cells with repeated UVB irradiation. Knockdown of miR4298 changed the expression of p-JNK and p-Erk by 1.708 ± 0.54 (*P* < 0.001) and 1.096 ± 0.7836 (*P* < 0.05), while p-p38 was not significantly changed (−0.002173 ± 0.058713, *P* > 0.05) (shown in Figure 4C).

The expression of Smad2 and TGF-β was changed after repeated UVB irradiation by −0.2330 ± 0.0568 (*P* < 0.0001) and 1.512 ± 0.056 (*P* < 0.0001), respectively. Overexpression of miR4298 in HaCaT cells decreased Smad2 and TGF-β by 0.1008 ± 0.0404 (*P* < 0.001) and 0.3072 ± 0.0763 (*P* < 0.0001) (shown in Figure 5B), whereas knockdown increased the Smad2 by 0.01039 ± 0.05687 and TGF-β by 1.713 ± 0.057 (*P* < 0.0001) in irradiated HaCaT cells (shown in Figure 5B).

### 3.5 Overexpression miR-4298 increased Cathepsin D expression

**Figure 6:**
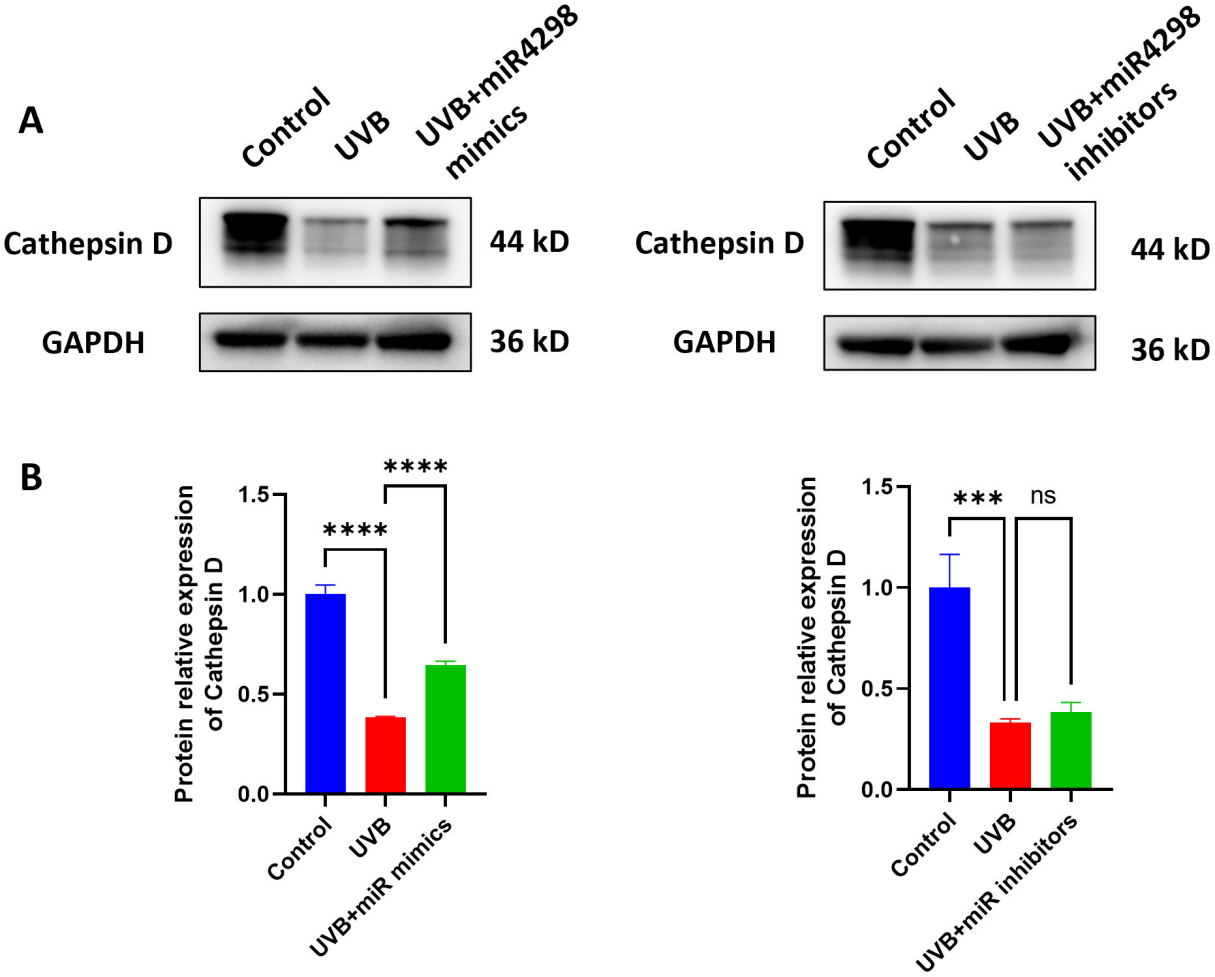
Effect of miR4298 on Cathepsin D protein expression. **(A)** Western blot results of Cathepsin D in different cell groups. **(B)** Statistical display of protein gray value (***P < 0.001, and ****P < 0.0001, ns: no significance).

After UVB radiation for 7 days, the expression of Cathepsin D in HaCaT cells was decreased by 0.6163 ± 0.075 (*P* < 0.0001) (shown in Figure 6A). Overexpression of miR4298 in HaCaT cells increased Cathepsin D protein level by 0.2617 ± 0.0749 (*P* < 0.0001), whereas knockdown decreased Cathepsin D by 0.05197 ± 0.24827 (shown in Figure 6B).

## 4 Discussion

Skin photoaging is closely related to repeated chronic ultraviolet radiation (31). UVB, which has shorter wavelengths and higher energy, not only primarily damages epidermal keratin-forming cells but also indirectly affects the dermis (32).

Recently, an increasing number of studies have revealed that miRNAs and lncRNAs are important regulators in skin photoaging. UV radiation can alter miRNA expression profiles (33). In addition, the miRNA-LncRNA-mRNA-protein regulatory network has gained recognition (29, 34, 35), and the concept of the “competitive endogenous RNA” (ceRNA) hypothesis has emerged. Many research studies indicated the existence of interactions between long non-coding RNAs (lncRNAs) and microRNAs (miRNAs) (36–38). LncRNAs, previously known as ceRNAs, can act as target mimics, “sponges,” or “decoys” for miRNAs (39). When miRNAs bind to lncRNAs, they not only change the expression of lncRNA itself but also regulate the expression of downstream target genes or proteins (13).

Further studies have revealed that MiR-551b-3p can act as a sponge, binding to lncPVT1, and delay the skin photoaging process by inhibiting the ERK/p38 MAPK signaling pathway (40). LncRNA H19 ameliorated UVB-induced skin photodamage by sponging adsorbing miR-138 and upregulating SIRT1 expression (41). MicroRNA-663a sponges bind to the lncRNA RP11-670E13.6 and delay UVB radiation-induced dermal fibroblast senescence by interacting with hnRNPH (42). Concerning HaCaT cells, the results of Meng et al. demonstrated that upregulated lncRNA-MSX2P1 promoted IL-22-stimulated cell growth through inhibition of miR-6731-5p, and they hypothesized that lncRNA-MSX2P1 functioned as an endogenous sponge that directly bound to miR-6731-5p (43). In another approach, exosomes from adipose-derived mesenchymal stem cells, highly enriched in the lncRNA FOXD2-AS1, promoted HaCaT cell migration and proliferation by downregulating miR-185-5p and upregulating ROCK2 (44). They found that lncRNAs can negatively target miRNAs, thereby affecting the function of HaCaT cells, and in our study, the changes in the expression of miR4298 and lncKRTAP5-6-3 were consistent. We found that overexpression of miR4298 can upregulate the expression of lncKRTAP5-6-3, and miR4298 inhibitors can downregulate the lncKRTAP5-6-3′s expression. Based on the above studies, we speculated that lncKRTAP5-6-3 could potentially act as a sponge for miR4298 in the UVB-induced photodamage process.

Studies have shown that abnormal accumulation of AGEs in photoaged skin was associated with the inhibition of cellular autophagy (45). Cathepsin D(CTSD) could delay the photoaging process by degrading excess AGEs (5, 8). Previous studies found that CTSD was highly expressed in psoriasis and traumatic wounds and suggested that CTSD played an important role in the epidermal barrier repair process and wound healing promotion (46). Our group previously explored the role of CTSD in skin photoaging and found that CTSD was negatively correlated with AGEs accumulation (8). In this study, we identified that overexpression of miR4298 could upregulate downstream CTSD expression which then inhibited chronic UVB damage in HaCaT cells. Our previous study suggested that lncKRTAP5-6-3 may act as an upstream regulator of changes in CTSD expression in chronically UV-damaged skin (12). In this study, we found that miR4298 upregulated the expression of lncKRTAP5-6-3 and CTSD in chronic UVB-damaged HaCaT cells. We therefore speculated that miR4298 could act as a sponge, binding to lncKRTAP5-6-3, which in turn upregulates the expression level of CTSD.

Various signaling pathways, including TGF-β, PI3K/AKT/mTOR, Nrf2, and MAPK signaling pathways, were involved in the development of skin photoaging (47–49). The mitogen-activated protein kinase (MAPK) signaling pathway is divided into three subfamilies: ERK, p38, and JNK (50). JNK and p38 subfamilies are mainly related to cell stress and apoptosis (51, 52). The ERK subfamily is the most widely studied signaling pathway of MAPK, which is closely related to cell proliferation and differentiation, and plays a role in the development of many diseases (53). Xu et al. found that WPH could slow down the process of photoaging in rat skin by regulating the MAPK/AP-1/MMP-1 and TGF-β/Smad signaling pathways (54). In HaCaT cells, several studies have shown that the MAPK signaling pathway plays a role in UVB-induced photoaging of these cells (55). A *Rosmarinus officinalis* extract reduced UVB-induced acute photodamage by inhibiting the MAPK/AP-1 signaling pathway (56). Isoquercitrin protects against UVB-induced photodamage in HaCaT cells through anti-inflammatory, antioxidant, and modulation of MAPK and JAK2-STAT3 pathways (57). Syringaresinol inhibits UVA-induced photodamage by suppressing MAPK/AP-1 signaling in HaCaT keratinocytes (58). Bamboo leaf flavonoids protect HaCaT cells from UVB photodamage by inhibiting p38 MAPK and autophagy signaling (59). In this study, we found that changes in p-ERK signaling were consistent with the intervention of miR4298 and suggested that miR4298 may regulate CTSD by modulating the MAPK/ERK signaling pathway. Although phosphorylation levels of p38 and JNK were also increased in HaCaT cells after UVB radiation, the upregulation of miR4298 had no significant effect on the expression level of p-p38. Moreover, the expression level of p-JNK was increased in both overexpression and knockdown of miR4298 compared to the UVB-damaged group, which is inconsistent with our scientific hypothesis.

Regarding the TGF-β/Smad2 pathway, the level of TGF-β expression in HaCaT cells increased after UVB radiation. Overexpression of miR4298 decreased the level of TGF-β expression, and accordingly, inhibition of miR4298 expression resulted in a higher level of TGF-β expression. However, when we detected the changes in Smad 2 expression, we found that UVB radiation decreased Smad 2 levels, indicating that miR4298 may not regulate the TGF-β/Smad2 pathway in UVB-induced photodamage in HaCaT cells. Transforming growth factor β (TGF-β) superfamily signaling plays an important role in the regulation of cell growth, differentiation, and development in numerous biological systems (60, 61). Its signaling molecules are Smad2 and Smad3 in the TGF-β/activin pathway and Smad1/5/9 in the bone morphogenetic protein (BMP) pathway. In addition, TGF-β signaling can also affect the Smad-independent pathway under certain circumstances (62–64).

Cathepsin D (CTSD) was negatively correlated with the accumulation of AGEs during skin photoaging. AGEs promote the generation of cellular reactive oxygen species (ROS), which triggers the activation of a number of cascade reactions and signaling pathways, thereby promoting inflammation and cellular aging (65). Studies have shown that AGEs influence the development of various diseases, such as diabetes and periodontitis, through the MAPK signaling pathway (66, 67). In the skin, RAGE, MAPK, and NF-κB pathways have been found to be involved in AGE-induced MMP-9 activation in HaCaT keratinocytes (68). In the present study, chronic UVB radiation led to activation of p-ERK signaling and downregulation of CTSD expression in HaCaT cells, which could potentially cause accumulation of AGEs and promote ROS generation, resulting in a decrease in cell number and acceleration of the UVB damage process. This is different from apoptosis because AGEs can be degraded by lysosomal proteases through cellular autophagy, and we suggest that UVB radiation downregulates the expression of CTSD through activation of the p-ERK pathway and is likely to ultimately affect the degradation of AGEs.

In this study, we used the HaCaT cell line for our experiments. It has been the first permanent epithelial cell line derived from adult human skin exhibiting largely normal epidermal differentiation under various conditions, although there are some differences in cell–cell or cell–matrix interactions and communicating pathways. While the majority of properties and functions of normal keratinocytes are retained, this cell line has a virtually infinitive life span but is *per se* not carcinogenic. HaCaT cells reveal a typical UVB-induced p53 point mutation being already present in the founder's skin (69). P Boukamp et al. found that spontaneous transformation of human adult keratinocytes can occur *in vitro* and is associated with sequential chromosomal variation (70). It is important to note that the epidermis is composed of several distinct layers, including the basal layer, spinous layer, granular layer, transparent layer, and stratum corneum, all of which are anchored to the dermis by the basement membrane (71). This includes the sequential expression, synthesis, and processing of differentiation-specific proteins, such as keratin, involucrin, and filaggrin, as well as profound cellular changes from basal cells to eventually very resistant squamous cells. Therefore, the skin condition *in vivo* is different from that of *in vitro* monolayer cell culture. Recently, an increasing number of studies have demonstrated that skin tissue structures can be simulated by 3D co-culture, where keratinocytes grow at the air–liquid interface on top of a skin fibroblast-loaded collagen gel (72–74). This allows cell-to-cell and cell–matrix interactions to occur between the two tissue compartments, mimicking the growth conditions of skin cells *in vivo*, which will be the direction of our future efforts. Our research focuses on the regulatory network of UVB, but solar UV radiation also includes UVA. UVA1, due to its longer wavelength, can penetrate the dermal layer of the skin, causing oxidative stress that affects both the epidermis and dermis. This leads to the production of reactive oxygen species (ROS), which in turn causes lipid peroxidation, protein changes, and the formation of DNA photoproducts (75). In the future, we will pay more attention to the different biological effects of UVA and UVB radiation.

In conclusion, this study focuses on the miR4298-LnckKRTAP563-CTSD regulatory network. We determined that miR4298 could upregulate LncKRTAP5-6-3 expression levels in chronic UVB-damaged HaCaT cells and could upregulate CTSD by inhibiting the ERK/MAPK signaling pathway. These findings may lay the experimental foundation for the development of new small-molecule RNA drugs and may provide new targets and ideas for the treatment and intervention of chronic ultraviolet-damaged (photoaging) skin and light-related dermatoses.

## Data Availability

The original contributions presented in the study are included in the article/supplementary material, further inquiries can be directed to the corresponding authors.

## References

[1] Berneburg M Plettenberg H Krutmann J. Photoaging of human skin. Photodermatol Photoimmunol Photomed. (2000) 16:239–44. 10.1034/j.1600-0781.2000.160601.x 11132125

[2] Li N Zhang K Mu X Tian Q Liu W Gao T. Astragalin attenuates Uvb radiation-induced actinic keratosis formation. Anticancer Agents Med Chem. (2018) 18:1001–8. 10.2174/1871520618666171229190835 29298652 PMC6327139

[3] Wolf Y Bartok O Patkar S Eli GB Cohen S Litchfield K. Uvb-induced tumor heterogeneity diminishes immune response in melanoma. Cell. (2019) 179:219–35.e21. 10.1016/j.cell.2019.08.032 31522890 PMC6863386

[4] Camponogara C Oliveira SM. Are Trpa1 and Trpv1 channel-mediated signalling cascades involved in Uvb radiation-induced sunburn? Environ Toxicol Pharmacol. (2022) 92:103836. 10.1016/j.etap.2022.103836 35248760

[5] Qu Y Wang M Lan J Huang X Huang J Li H. Circrna-406918 enhances the degradation of advanced glycation end products in photoaged human dermal fibroblasts via targeting cathepsin D. Photodermatol Photoimmunol Photomed. (2023) 39:487–97. 10.1111/phpp.12887 37253092

[6] Grimm S Ernst L Grötzinger N Höhn A Breusing N Reinheckel T. Cathepsin D is one of the major enzymes involved in intracellular degradation of age-modified proteins. Free Radic Res. (2010) 44:1013–26. 10.3109/10715762.2010.495127 20560835

[7] Minarowska A Gacko M Karwowska A Minarowski Ł. Human cathepsin D. Folia Histochem Cytobiol. (2008) 46:23–38. 10.2478/v10042-008-0003-x 18296260

[8] Xu X Zheng Y Huang Y Chen J Gong Z Li Y. Cathepsin D contributes to the accumulation of advanced glycation end products during photoaging. J Dermatol Sci. (2018) 90:263–75. 10.1016/j.jdermsci.2018.02.009 29501392

[9] Zheng Y Chen H Lai W Xu Q Liu C Wu L. Cathepsin D repairing role in photodamaged skin barrier. Skin Pharmacol Physiol. (2015) 28:97–102. 10.1159/000363248 25402676

[10] Khan I Maldonado E Vasconcelos V O'Brien SJ Johnson WE Antunes A. Mammalian keratin associated proteins (krtaps) subgenomes: disentangling hair diversity and adaptation to terrestrial and aquatic environments. BMC Genomics. (2014) 15:779. 10.1186/1471-2164-15-779 25208914 PMC4180150

[11] Berens EB Sharif GM Schmidt MO Yan G Shuptrine CW Weiner LM. Keratin-associated protein 5-5 controls cytoskeletal function and cancer cell vascular invasion. Oncogene. (2017) 36:593–605. 10.1038/onc.2016.234 27375028 PMC5215748

[12] Zheng Y Xu Q Peng Y Gong Z Chen H Lai W. Expression profiles of long noncoding RNA in Uva-induced human skin fibroblasts. Skin Pharmacol Physiol. (2017) 30:315–23. 10.1159/000477972 29069654

[13] Panni S Lovering RC Porras P Orchard S. Non-coding RNA regulatory networks. Biochim Biophys Acta Gene Regul Mech. (2020) 1863:194417. 10.1016/j.bbagrm.2019.194417 31493559

[14] Xiao S Zhou Y Liu Q Zhang T Pan D. Identification of pivotal micrornas and target genes associated with persistent atrial fibrillation based on bioinformatics analysis. Comput Math Methods Med. (2021) 2021:6680211. 10.1155/2021/6680211 33747117 PMC7960048

[15] Wang ZQ Zhang MY Deng ML Weng NQ Wang HY Wu SX. Low serum level of Mir-485-3p predicts poor survival in patients with glioblastoma. PLoS ONE. (2017) 12:e0184969. 10.1371/journal.pone.0184969 28931080 PMC5607158

[16] 杜经柱, 陈奇, 尹浩, 李锋. Mir-4298 靶向 tgi…细胞的增. 殖和迁移的机制研究. 肿瘤代谢与营养电子杂志 (2020) 7:225–30.

[17] Chen X Lu P Wang DD Yang SJ Wu Y Shen HY. The role of mirnas in drug resistance and prognosis of breast cancer formalin-fixed paraffin-embedded tissues. Gene. (2016) 595:221–6. 10.1016/j.gene.2016.10.015 27746365

[18] An J Pan Y Yan Z Li W Cui J Yuan J. et al. Mir-23a in amplified 19p1313 loci targets metallothionein 2a and promotes growth in gastric cancer cells. J Cell Biochem. (2013) 114:2160–9. 10.1002/jcb.24565 23553990

[19] Yu Y Zhang X Liu F Zhu P Zhang L Peng Y. A stress-induced Mir-31-clock-erk pathway is a key driver and therapeutic target for skin aging. Nat Aging. (2021) 1:795–809. 10.1038/s43587-021-00094-8 37117623

[20] Gao W Yuan LM Zhang Y Huang FZ Gao F Li J. Mir-1246-overexpressing exosomes suppress Uvb-induced photoaging via regulation of Tgf-β/smad and attenuation of Mapk/Ap-1 pathway. Photochem Photobiol Sci. (2023) 22:135–46. 10.1007/s43630-022-00304-1 36114328

[21] Wang S Zeng Y Zhou JM Nie SL Peng Q Gong J. Microrna-1246 promotes growth and metastasis of colorectal cancer cells involving Ccng2 reduction. Mol Med Rep. (2016) 13:273–80. 10.3892/mmr.2015.4557 26573378

[22] Dai YC Pan Y Quan MM Chen Q Pan Y Ruan YY. Microrna-1246 mediates drug resistance and metastasis in breast cancer by targeting Nfe2l3. Front Oncol. (2021) 11:677168. 10.3389/fonc.2021.677168 34926237 PMC8671458

[23] Wu L Zuo N Pan S Wang Y Wang Q Ma J. The exosomal Mir-1246 of laryngeal squamous cell carcinoma induces polarization of M2 type macrophages and promotes the invasiveness of laryngeal squamous cell carcinoma. J Oncol. (2022) 2022:4424221. 10.1155/2022/4424221 36199785 PMC9529393

[24] Wu L Zuo N Pan S Wang Y Wang Q Ma J. Mir-1246 promotes laryngeal squamous cell carcinoma progression by interacting with Thbs1. J Environ Pathol Toxicol Oncol. (2022) 41:65–75. 10.1615/JEnvironPatholToxicolOncol.2022040516 35993956

[25] Chai S Ng KY Tong M Lau EY Lee TK Chan KW. Octamer 4/microrna-1246 signaling axis drives Wnt/β-catenin activation in liver cancer stem cells. Hepatology. (2016) 64:2062–76. 10.1002/hep.28821 27639189

[26] Huang JL Fu YP Gan W Liu G Zhou PY Zhou C. Hepatic stellate cells promote the progression of hepatocellular carcinoma through microrna-1246-Rorα-Wnt/β-catenin axis. Cancer Lett. (2020) 476:140–51. 10.1016/j.canlet.2020.02.012 32061951

[27] Yang F Xiong H Duan L Li Q Li X Zhou Y. Mir-1246 Promotes metastasis and invasion of A549 cells by targeting Gsk-3β-mediated Wnt/β-catenin pathway. Cancer Res Treat. (2019) 51:1420–9. 10.4143/crt.2018.638 30913872 PMC6790833

[28] Deng Y Phillips K Feng ZP Smith PN Li RW. Aseptic loosening around total joint replacement in humans is regulated by Mir-1246 and Mir-6089 Via the Wnt signalling pathway. J Orthop Surg Res. (2024) 19:94. 10.1186/s13018-024-04578-2 38287447 PMC10823634

[29] Lin Y Lin M Liu Y Zhang J Lai W Xu Q. Predicting Mirna-Lncrna-Mrna network in ultraviolet a-induced human skin photoaging. J Cosmet Dermatol. (2021) 20:1875–84. 10.1111/jocd.13760 33025709

[30] Lin Y Sun Y Hou W Chen X Zhou F Xu Q. Fto-mediated regulation of M6a methylation is closely related to apoptosis induced by repeated Uv irradiation. J Dermatol Sci. (2024) 114:124–32. 10.1016/j.jdermsci.2024.01.001 38749796

[31] Salminen A Kaarniranta K Kauppinen A. Photoaging: Uv Radiation-induced inflammation and immunosuppression accelerate the aging process in the skin. Inflamm Res. (2022) 71:817–31. 10.1007/s00011-022-01598-8 35748903 PMC9307547

[32] Garssen J Norval M. el-Ghorr A, Gibbs NK, Jones CD, Cerimele D, et al. Estimation of the effect of increasing Uvb exposure on the human immune system and related resistance to infectious diseases and tumours. J Photochem Photobiol B. (1998) 42:167–79. 10.1016/S1011-1344(97)00122-X 9595706

[33] Srivastava A Karlsson M Marionnet C Bernerd F Gueniche A Rawadi CEL. Identification of chronological and photoageing-associated micrornas in human skin. Sci Rep. (2018) 8:12990. 10.1038/s41598-018-31217-8 30154427 PMC6113407

[34] Soheilifar MH Masoudi-Khoram N Shirkavand A Ghorbanifar S. Non-coding Rnas in photoaging-related mechanisms: a new paradigm in skin health. Biogerontology. (2022) 23:289–306. 10.1007/s10522-022-09966-x 35587318

[35] Kim S. Lncrna-Mirna-Mrna regulatory networks in skin aging and therapeutic potentials. Front Physiol. (2023) 14:1303151. 10.3389/fphys.2023.1303151 37881693 PMC10597623

[36] Cesana M Cacchiarelli D Legnini I Santini T Sthandier O Chinappi M. A long noncoding RNA controls muscle differentiation by functioning as a competing endogenous RNA. Cell. (2011) 147:358–69. 10.1016/j.cell.2011.10.031 22000014 PMC3234495

[37] Salmena L Poliseno L Tay Y Kats L Pandolfi PP A. Cerna hypothesis: the rosetta stone of a hidden RNA language? Cell. (2011) 146:353–8. 10.1016/j.cell.2011.07.014 21802130 PMC3235919

[38] Tay Y Rinn J Pandolfi PP. The multilayered complexity of cerna crosstalk and competition. Nature. (2014) 505:344–52. 10.1038/nature12986 24429633 PMC4113481

[39] Yamamura S Imai-Sumida M Tanaka Y Dahiya R. Interaction and cross-talk between non-coding RNAs. Cell Mol Life Sci. (2018) 75:467–84. 10.1007/s00018-017-2626-6 28840253 PMC5765200

[40] Tang H Xiong Q Yin M Feng H Yao F Xiao X. Lncrna Pvt1 delays skin photoaging by sequestering Mir-551b-3p to release Aqp3 expression via cerna mechanism. Apoptosis. (2023) 28:912–24. 10.1007/s10495-023-01834-4 37000315

[41] Gao W Zhang Y Yuan L Huang F Wang YS. Long non-coding Rna H19-overexpressing exosomes ameliorate Uvb-induced photoaging by upregulating Sirt1 via sponging Mir-138. Photochem Photobiol. (2023) 99:1456–67. 10.1111/php.13801 36916469

[42] Li M Li L Zhang X Zhao H Wei M Zhai W. et al. Lncrna Rp11-670e136, interacted with Hnrnph, delays cellular senescence by sponging microrna-663a in Uvb damaged dermal fibroblasts. Aging (Albany NY). (2019) 11:5992–6013. 10.18632/aging.102159 31444317 PMC6738423

[43] Qiao M Li R Zhao X Yan J Sun Q. Up-regulated Lncrna-Msx2p1 promotes the growth of Il-22-stimulated keratinocytes by inhibiting Mir-6731-5p and activating S100a7. Exp Cell Res. (2018) 363:243–54. 10.1016/j.yexcr.2018.01.014 29339075

[44] Chang H Chen J Ding K Cheng T Tang S. Highly-expressed Lncrna Foxd2-As1 in adipose mesenchymal stem cell derived exosomes affects hacat cells via regulating Mir-185-5p/Rock2 axis. Adipocyte. (2023) 12:2173513. 10.1080/21623945.2023.2173513 36775902 PMC9928455

[45] Huang Y Li Y Qu Y Zheng Y Ouyang M Zhang Y. Uva-induced photoaging inhibits autophagic degradation by impairing lysosomal function in dermal fibroblasts. Biochem Biophys Res Commun. (2019) 518:611–8. 10.1016/j.bbrc.2019.08.103 31445710

[46] Egberts F Heinrich M Jensen JM Winoto-Morbach S Pfeiffer S Wickel M. Cathepsin D is involved in the regulation of transglutaminase 1 and epidermal differentiation. J Cell Sci. (2004) 117:2295–307. 10.1242/jcs.01075 15126630

[47] Li Y Song P He J Liu B Liu S Zhou Y. Comparison between injectable platelet-rich fibrin and platelet-rich plasma in ameliorating Uva-induced photoaging in human dermal fibroblasts Via the activation of Tgf-β/Smad signaling pathway. Photochem Photobiol. (2022) 98:1395–401. 10.1111/php.13628 35365859

[48] Kotb EA El-Shiekh RA Abd-Elsalam WH El Sayed N El Tanbouly N El Senousy AS. Protective potential of frankincense essential oil and its loaded solid lipid nanoparticles against Uvb-induced photodamage in rats via Mapk and Pi3k/Akt signaling pathways; a promising anti-aging therapy. PLoS ONE. (2023) 18:e0294067. 10.1371/journal.pone.0294067 38127865 PMC10735031

[49] Liu W Yan F Xu Z Chen Q Ren J Wang Q. Urolithin a protects human dermal fibroblasts from Uva-induced photoaging through Nrf2 activation and mitophagy. J Photochem Photobiol B. (2022) 232:112462. 10.1016/j.jphotobiol.2022.112462 35567884

[50] Sun Y Liu WZ Liu T Feng X Yang N Zhou HF. Signaling pathway of Mapk/Erk in cell proliferation, differentiation, migration, senescence and apoptosis. J Recept Signal Transduct Res. (2015) 35:600–4. 10.3109/10799893.2015.1030412 26096166

[51] Garg R Kumariya S Katekar R Verma S Goand UK Gayen JR. Jnk signaling pathway in metabolic disorders: an emerging therapeutic target. Eur J Pharmacol. (2021) 901:174079. 10.1016/j.ejphar.2021.174079 33812885

[52] Zarubin T Han J. Activation and signaling of the P38 map kinase pathway. Cell Res. (2005) 15:11–8. 10.1038/sj.cr.7290257 15686620

[53] Guo YJ Pan WW Liu SB Shen ZF Xu Y Hu LL. Erk/Mapk signalling pathway and tumorigenesis. Exp Ther Med. (2020) 19:1997–2007. 10.3892/etm.2020.8454 32104259 PMC7027163

[54] Xu D Li C Zhao M. Attenuation of Uv-induced skin photoaging in rats by walnut protein hydrolysates is linked to the modulation of Mapk/Ap-1 and Tgf-β/Smad signaling pathways. Food Funct. (2022) 13:609–23. 10.1039/D1FO02598H 34927661

[55] Kim M Park YG Lee HJ Lim SJ Nho CW. Youngiasides A and C isolated from youngia denticulatum inhibit Uvb-induced Mmp expression and promote type I procollagen production via repression of Mapk/Ap-1/Nf-κb and activation of Ampk/Nrf2 in hacat cells and human dermal fibroblasts. J Agric Food Chem. (2015) 63:5428–38. 10.1021/acs.jafc.5b00467 25994852

[56] Fang M Lee HM Oh S Zheng S Bellere AD Kim M. Rosa davurica inhibits skin photoaging via regulating Mapk/Ap-1, Nf-κb, and Nrf2/Ho-1 signaling in Uvb-irradiated hacats. Photochem Photobiol Sci. (2022) 21:2217–30. 10.1007/s43630-022-00290-4 36103110

[57] Li Y Ma Y Yao Y Ru G Lan C Li L. Protective effect of isoquercitrin on Uvb-induced injury in hacat cells and mice skin through anti-inflammatory, antioxidant, and regulation of Mapk and Jak2-Stat3 pathways. Photochem Photobiol. (2024) 100:1507–18. 10.1111/php.13919 38337181

[58] Oh JH Joo YH Karadeniz F Ko J Kong CS. Syringaresinol inhibits Uva-induced Mmp-1 expression by suppression of Mapk/Ap-1 signaling in hacat keratinocytes and human dermal fibroblasts. Int J Mol Sci. (2020) 21:e21113981. 10.3390/ijms21113981 32492931 PMC7312901

[59] Gu Y Xue F Xiao H Chen L Zhang Y. Bamboo leaf flavonoids suppress oxidative stress-induced senescence of hacat cells and Uvb-induced photoaging of mice through P38 Mapk and autophagy signaling. Nutrients. (2022) 14:e14040793. 10.3390/nu14040793 35215447 PMC8876272

[60] Horbelt D Denkis A Knaus PA. Portrait of transforming growth factor B Superfamily signalling: background matters. Int J Biochem Cell Biol. (2012) 44:469–74. 10.1016/j.biocel.2011.12.013 22226817

[61] Schmierer B Hill CS. Tgfbeta-Smad signal transduction: molecular specificity and functional flexibility. Nat Rev Mol Cell Biol. (2007) 8:970–82. 10.1038/nrm2297 18000526

[62] Derynck R Zhang YE. Smad-dependent and Smad-independent pathways in Tgf-Beta family signalling. Nature. (2003) 425:577–84. 10.1038/nature02006 14534577

[63] Kitisin K Saha T Blake T Golestaneh N Deng M Kim C. Tgf-Beta signaling in development. Sci STKE. (2007) 2007:cm1. 10.1126/stke.3992007cm1 17699101

[64] Ikushima H Miyazono K. Tgfbeta signalling: a complex web in cancer progression. Nat Rev Cancer. (2010) 10:415–24. 10.1038/nrc2853 20495575

[65] Wan L Bai X Zhou Q Chen C Wang H Liu T. The advanced glycation end-products (Ages)/Ros/Nlrp3 inflammasome axis contributes to delayed diabetic corneal wound healing and nerve regeneration. Int J Biol Sci. (2022) 18:809–25. 10.7150/ijbs.63219 35002527 PMC8741862

[66] Li JS Ji T Su SL Zhu Y Chen XL Shang EX. Mulberry leaves ameliorate diabetes via regulating metabolic profiling and ages/rage and P38 Mapk/Nf-κb pathway. J Ethnopharmacol. (2022) 283:114713. 10.1016/j.jep.2021.114713 34626776

[67] Yi X Song Y Xu J Wang L Liu L Huang D. Nlrp10 promotes ages-induced Nlrp1 and Nlrp3 inflammasome activation via Ros/Mapk/Nf-κb signaling in human periodontal ligament cells. Odontology. (2024) 112:100–11. 10.1007/s10266-023-00813-0 37043073

[68] Zhu P Ren M Yang C Hu YX Ran JM Yan L. Involvement of rage, Mapk and Nf-κb pathways in ages-induced Mmp-9 activation in hacat keratinocytes. Exp Dermatol. (2012) 21:123–9. 10.1111/j.1600-0625.2011.01408.x 22229442

[69] Lehman TA Modali R Boukamp P Stanek J Bennett WP Welsh JA. P53 Mutations in human immortalized epithelial cell lines. Carcinogenesis. (1993) 14:833–9. 10.1093/carcin/14.5.833 8504475

[70] Boukamp P Petrussevska RT Breitkreutz D Hornung J Markham A Fusenig NE. Normal keratinization in a spontaneously immortalized aneuploid human keratinocyte cell line. J Cell Biol. (1988) 106:761–71. 10.1083/jcb.106.3.761 2450098 PMC2115116

[71] Arda O Göksügür N Tüzün Y. Basic histological structure and functions of facial skin. Clin Dermatol. (2014) 32:3–13. 10.1016/j.clindermatol.2013.05.021 24314373

[72] Andrade TAM da Silva VA Scheck K Garay T Sharma R Willerth SM. 3D bioprinting a novel skin co-culture model using human keratinocytes and fibroblasts. J Biomed Mater Res A. (2024). 113:e37831. 10.1002/jbm.a.37831 39487730

[73] Rademacher F Simanski M Gläser R Harder J. Skin microbiota and human 3D skin models. Exp Dermatol. (2018) 27:489–94. 10.1111/exd.13517 29464787

[74] Kang MS Jang J Jo HJ Kim WH Kim B Chun HJ. Advances and innovations of 3D bioprinting skin. Biomolecules. (2022) 13:e10055. 10.3390/biom13010055 36671440 PMC9856167

[75] Bernerd F Passeron T Castiel I Marionnet C. The damaging effects of long Uva (Uva1) rays: a major challenge to preserve skin health and integrity. Int J Mol Sci. (2022) 23:8243. 10.3390/ijms23158243 35897826 PMC9368482

